# Tibial periosteal distraction for ischemic leg ulcers: animal and clinical cohort study

**DOI:** 10.1186/s12916-025-04586-x

**Published:** 2025-12-28

**Authors:** Peilin Zhou, Wenqiang Wang, Yi You, Nian Zhou, Taifeng Zhang, Jiangtao Yang, Xiang Chen, Linghui Tang, Meng Gan, Wei Xu, Wengao Wu, Boyang Liu, Huiyan Liu, Xiaochong Zou, Yongzhen Liu, Xiao Peng, Lu Wei, Xingyu Chen, Feng Yang, Yueyue Zhu, Ruibin Feng, Xiaoping Yu, Junliang Ye, Ronghan Wu, Yu Chen, Lan Lan, Jiajun Chen, Yi Ding, Xinyu Nie, Bingfeng Lu, Naxin Zeng, Qikai Hua

**Affiliations:** 1 Department of Bone and Joint Surgery, Guangxi Diabetic Foot Salvage Engineering Research Center/Research Centre for Regenerative Medicine, The First Affiliated Hospital of Guangxi Medical University, No. 6 Shuangyong Road, Nanning, Guangxi 530021 China; 2 Collaborative Innovation Centre of Regenerative Medicine and Medical BioResource Development and Application Co-Constructed By the Province and Ministry, Guangxi Medical University, 22 Shuangyong Road, Nanning, Guangxi 530021 China; 3 Department of Cardiovascular Surgery, Yueyang Central Hospital, No. 39, Dongmaoling Road, Yueyanglou District, Yueyang, Hunan 414000 China; 4 Department of Radiology, The Second Affiliated Hospital of Guangxi Medical University, No. 166 Daxue East Road, Nanning, Guangxi 530021 China; 5 Department of Orthopedics, Qinzhou Second People’s Hospital, No. 219, Wenfeng South Road, Qinbei District, Qinzhou City, Guangxi Zhuang Autonomous Region 535000 China; 6 School of Nursing, Guangxi Medical University, 22 Shuangyong Road, Nanning, Guangxi 530021 China; 7 Department of Orthopedics, Yueyang Central Hospital, No. 39, Dongmaoling Road, Yueyanglou District, Yueyang, Hunan 414000 China; 8 Department of Radiology, The First Affiliated Hospital of Guangxi University of Chinese Medicine, Dongge Road, Qingxiu District, Nanning, Guangxi 530023 China; 9 Department of Orthopedics, The First Affiliated Hospital of USTC, Division of Life Sciences and Medicine, University of Science and Technology of China, 17 Lujiang Road, Luyang District, Hefei, Anhui 230002 China

**Keywords:** Tibial periosteal distraction, Ischemic leg ulcer, Chronic limb threatening ischemia, Diabetic foot ulcer, Tibial transverse transport

## Abstract

**Background:**

Ischemic leg ulcers (ILU) represent a severe manifestation of chronic limb-threatening ischemia (CLTI), characterized by high recurrence and amputation rates. This study evaluated the efficacy and safety of tibial periosteal distraction (TPD) for treating ILU through a combination of animal experiments and clinical trials.

**Methods:**

Nine Beagle dogs were randomly allocated into TPD group, tibial soft tissue distraction (TSD) group, and control group. Standardized 15-mm circular wounds were created on the foot, followed by periosteal distraction at a rate of 0.5 mm/day for 11 days. Parameters assessed included wound healing rates, serum VEGF levels, histopathological changes, and CT angiography/perfusion parameters. A multicenter retrospective cohort study was conducted from June 2019 to January 2024, enrolling 103 ILU patients treated with TPD compared with 127 patients receiving conventional treatment. Primary endpoints included ulcer healing rates at 3 and 6 months, amputation rates, and recurrence within 1 year. Secondary endpoints comprised complications and safety assessments.

**Results:**

In animal study, the TPD group demonstrated accelerated wound healing compared to both control and TSD groups, with residual wound area of 2.08 ± 1.68% on day 16, versus 5.46 ± 1.98% in the control group and 12.49 ± 2.97% in the TSD group. Serum VEGF levels were markedly elevated in the TPD group from days 8 to 16 (peak: 27.25 ± 2.16 pg/ml vs. 17.90 ± 1.72 pg/ml in controls, *P* < 0.01). CT angiography revealed enhanced collateral circulation in the distraction region with increased tissue perfusion parameters (equivalent blood volume and arterial flow). In clinical study, the TPD group achieved superior healing rates compared to controls: 90.3% vs. 77.2% at 3 months (*P* = 0.008) and 94.2% vs. 85.8% at 6 months (*P* = 0.039). Major amputation rate was reduced in the TPD group (2.9% vs. 9.5%, *P* = 0.046), while minor amputation rates showed no significant difference (38.8% vs. 44.1%, *P* = 0.421). Recurrence within 1 year was markedly decreased (8.7% vs. 19.7%, *P* = 0.020). Complications included pin tract infection in 7 cases (6.8%) and pain intolerance requiring distraction rate adjustment in 9 cases (8.7%), all successfully managed with conservative treatment.

**Conclusions:**

TPD offers a promising therapeutic option for CLTI patients, particularly those unsuitable for vascular reconstruction. Further prospective randomized trials are warranted to establish standardized protocols and optimize treatment parameters.

**Graphical Abstract:**

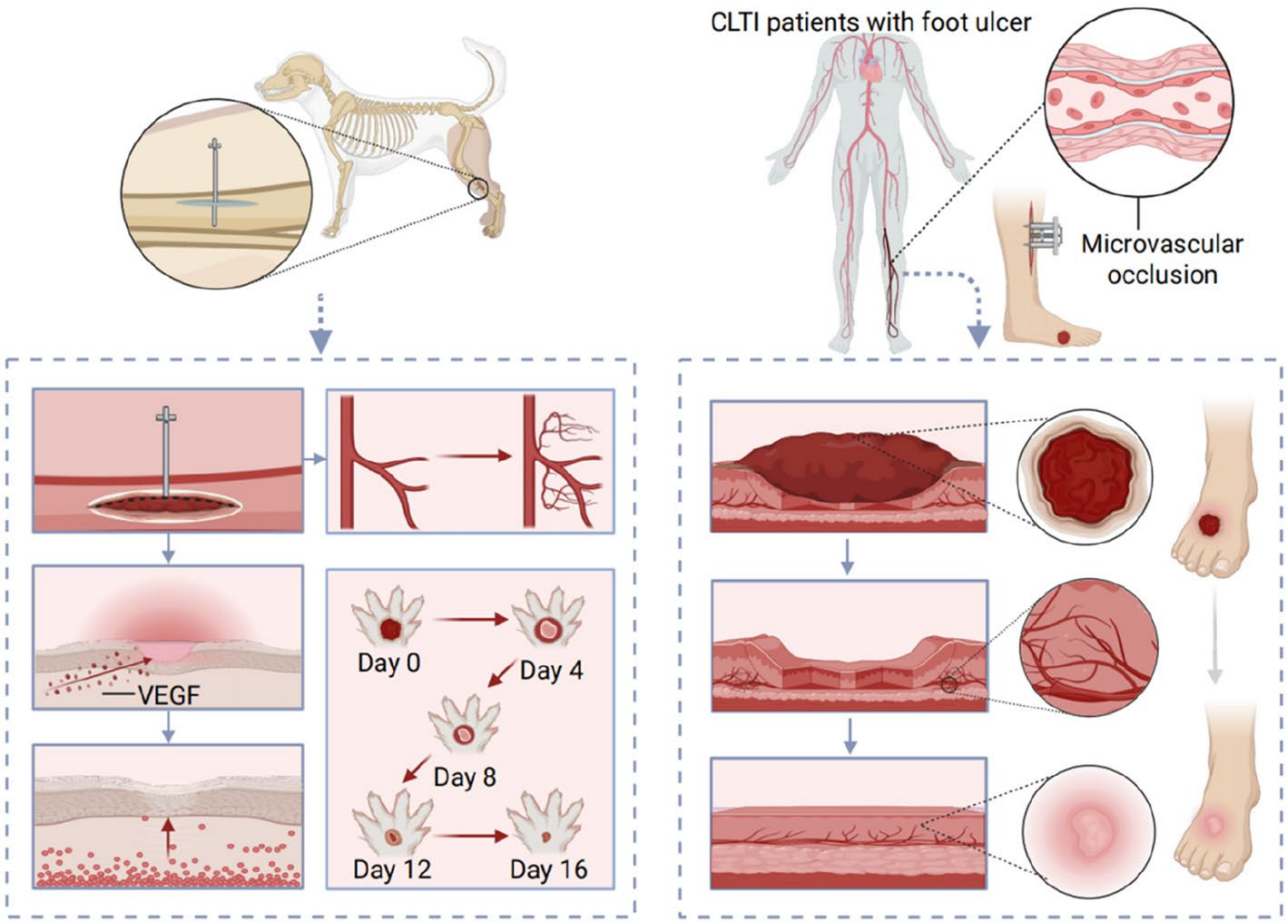

## Background

Ischemic leg ulcers (ILU) are a common type of chronic wound, typically caused by insufficient arterial blood supply to the lower extremities, particularly prevalent in patients with underlying diseases such as peripheral arterial disease (PAD) and diabetes. The pathophysiological characteristics include persistent tissue hypoperfusion, leading to insufficient oxygen and nutrient supply, accumulation of metabolic waste products, and ultimately resulting in tissue necrosis and ulcer formation. Treatment of ILU is challenging and often accompanied by prolonged slow healing and recurrent episodes, severely affecting patients’ quality of life. The global number of PAD patients exceeded 100 million in 2019 [1, 2]. Meanwhile, there are approximately 537 million diabetes patients worldwide, of whom 19–34% will develop foot ulcers during their lifetime, with about 70% of these ulcers involving ischemic factors [3, 4].

The refractory nature of ILU represents the main clinical challenge. Traditional treatment methods include vascular reconstruction surgery, pharmacological therapy, local wound care, hyperbaric oxygen therapy, physical rehabilitation, and lifestyle interventions [5]. Clinicians typically need to develop treatment plans based on patients’ individual characteristics, ulcer severity, types of vascular lesions, and overall health status, often employing multimodal combination therapy to optimize clinical outcomes [6]. However, even with these comprehensive treatment approaches, chronic ischemia and resulting non-healing wounds still lead to severe amputation for 3.6 to 68.4 people per 100,000 globally each year [7, 8]. More concerningly, approximately 40% of diabetic foot ulcer (DFU) patients experience recurrence within one year after ulcer healing [4]. The 5-year overall survival rate for ILU patients is only 50–60%, which is lower than that of many malignant tumor patients, demonstrating the severity of this disease and the grimness of its prognosis [9].

The Ilizarov technique is a bone lengthening and reconstruction technology developed by Soviet physician Gavriil Ilizarov in the 1950s. This technique is based on the “tension-stress effect” principle, using external fixation scaffolds to apply slow, continuous traction to bone tissue, promoting regeneration of bone tissue and surrounding soft tissues [10]. This technique was initially used to treat non-union fractures, bone defects, and limb deformities. With the development of the Ilizarov technique, tibial transverse transport (TTT) was proposed as an improved technique, improving local blood circulation and promoting wound healing through transverse bone tissue traction [11]. After ten years of development, the effectiveness of TTT has been proven by multiple institutions [12]. Despite the proven efficacy of TTT in clinical practice, researchers continue to pursue more simplified surgical approaches. The periosteum, a dense fibrous connective tissue membrane covering the bone surface, has an inner layer rich in vascular networks, osteoblasts, and mesenchymal stem cells, and possesses strong tissue regeneration and angiogenesis potential [13]. Under physiological and pathological conditions, the periosteum not only plays a key regulatory role in fracture repair and bone tissue reconstruction but is also an important tissue source for promoting local angiogenesis. Based on the tension-stress effect, tibial periosteal distraction (TPD) may achieve similar therapeutic effects to TTT in improving lower limb blood circulation by mechanically stimulating angiogenesis-related signaling pathways through mechanical stimulation. Gan et al. previously reported preliminary outcomes of TPD in a small single-center case series without a control group [14]. Building on this work, the present study combines animal experiments and multicenter clinical research to systematically evaluate the efficacy and safety of TPD in treating ILU. Additionally, we aimed to establish standardized surgical protocols to provide a safe and effective treatment option for lower limb ischemic diseases.

## Methods

### Animal selection and group assignment

Beagle dogs (Huainan Chenlang Experimental Animal Sales Co., Ltd., China) were selected as experimental animals, aged 2–3 years, male, weighing 15–19 kg. In accordance with the 3Rs principle, we aim to minimize the number of animals used, while ensuring that experimental differences are observable and that statistical power requirements for replication are met. A total of 9 dogs were randomly divided into TPD group, tibial soft tissue distraction (TSD) group, and control group, with 3 dogs in each group. In the TPD group, the periosteal distraction plate was installed beneath the periosteum; in the TSD group, the distraction plate was installed above the periosteum and beneath the soft tissue; both groups received the same care and adjustments. The control group did not have periosteal distraction devices installed.

### Animal care and monitoring

All dogs were housed in a conventional (non-barrier) facility at the Laboratory Animal Center of Guangxi Medical University. Animals were fed twice daily with standard dog chow and had ad libitum access to water. Post-operatively, daily dog walking time was increased to 1 h starting from the first day to allow energy expenditure and reduce the probability of biting or moving the scaffold, while avoiding vigorous activity that could cause the distraction plate to perforate the periosteum or skin. Dressing changes under anesthesia, blood sampling, and wound photography were performed on post-operative days 0, 4, 8, 12, and 16. No humane endpoints were established. All dogs were rehomed through animal welfare organizations after study completion.

### Animal specimen collection

Image J software was used to measure and analyze wounds at various time points, with each sample measured three times and averaged, followed by calculation of wound repair degree. Wound area was presented as a percentage: Wound area percentage = Current wound area/Original wound area × 100%. On post-operative day 16, wounds were in a near-healing state. After photographic documentation was completed, surgical wound sampling was performed. Arterial blood (5 ml) was collected from the femoral artery of anesthetized dogs in lateral position using a 22G needle. Blood samples were allowed to clot at 4 °C for 2 h, then centrifuged at 3000 rpm for 10 min at 4 °C. Serum was aliquoted (100 μl/tube) and stored at − 80 °C, avoiding repeated freeze–thaw cycles.

### Tissue embedding, sectioning, and staining

Obtained tissue specimens were immediately placed in 10% neutral buffered formalin solution (pH 7.2–7.4) and fixed at 4 °C for 24 h. Fixed tissues underwent gradient ethanol (70%, 80%, 90%, 95%, 100%) dehydration using an automatic dehydrator, with each step lasting 1 h. Subsequently, tissues were clarified in xylene twice, each for 1 h, then infiltrated with 58–60 °C paraffin 3 times, each for 1.5 h. After embedding completion, tissues were cut into 4 μm thick sections using a rotary microtome, placed on APES-treated slides, and dried in a 60 °C oven for 1 h. After drying, HE and Masson staining were performed, respectively.

### ELISA experiment

Enzyme-linked immunosorbent assay (ELISA) was used to detect blood vascular endothelial growth factor (VEGF) concentration (Cosmo Bio Technology Co., Ltd., China). First, standards (S0–S7) were prepared through serial dilution, and working solutions of wash buffer (1:25 dilution), biotin-labeled antibody working solution, and horseradish peroxidase-labeled avidin working solution (both 1:100 dilution) were prepared. After equilibrating all reagents at room temperature (25 °C) for 30 min, 50 μl of standard or sample was added to each well and incubated at 37 °C for 2 h; after discarding well contents, 100 μl of biotin-labeled antibody working solution was added and incubated for 1 h; plates were washed 3 times (200 μl/well, soaking for 2 min); 100 μl of horseradish peroxidase-labeled avidin working solution was added and incubated for 1 h; after washing plates 5 times, 90 μl of substrate solution was added and color development was performed at 37 °C in darkness for 15–30 min; finally, 50 μl of stop solution was added, and optical density values (OD values) of each well were measured at 450 nm wavelength within 5 min. Data were processed using standard curve analysis software to obtain final sample concentration results.

### Imaging evaluation

Before CT scan, each dog was anesthetized and fixed on a board in supine position and properly covered with a bandage on the legs to minimize motion. All CT perfusion (CTP) and CT angiography (CTA) examinations in this study were acquired using a 320-detector row CT scanner (Aquilion ONE scanner, Canon Medical Systems, Japan). Iodinated contrast medium (350 mgI/mL, 1.5mL/kg) was administered via the forelimb radial vein using a high-pressure injector: 4.5 mL/s for CTP and 4.0 mL/s for CTA. To minimize contrast carryover effects, a 5-minute delay was observed between CTP and subsequent CTA acquisitions. Imaging protocols were standardized as follows: CTP: 100 kV tube voltage, 55 mAs tube current, 0.5 mm slice thickness, range of 299mm; CTA: 120 kV tube voltage, 235 mAs tube current, 0.5 mm slice thickness. CT scanning (0.5 s/rotation) was started with a stationary rotation at the lower limbs, 7s after the beginning of the contrast medium injection followed by perfusion scanning (0.275s/rotation) at 14.3s, thereafter in 4.9s intervals. Perfusion data were dynamically acquired 17 times over 62.8 seconds, with time-attenuation curves automatically generated for arterial input function quantification. Tissue perfusion maps were derived using a single-compartment model. The scanning method for clinical patients references previous article [15].

All datasets were reconstructed on a Vitrea Advanced Visualization workstation (Canon Medical Systems, Japan). Perfusion analysis utilized CT Single-Input Body Perfusion 4D software (Version 7.6) with Patlak plot modeling, generating parametric maps of: Equivalent blood volume (EBV, mL/100 mL), Arterial flow (AF, mL/min/100 mL). CTA post-processing included volume rendering and maximum intensity projection reconstructions performed via Runoff angiography analysis software.

### Clinical patient grouping

To evaluate the clinical therapeutic effect of TPD on ILU, we conducted a multicenter retrospective real-world cohort study. Clinical patients were divided into TPD group and control group based on whether they received TPD treatment. Regardless of whether they were in the TPD group or control group, they could receive wound management according to their personal preferences. Wound management includes but is not limited to debridement, negative pressure wound therapy, bone cement, skin grafting, etc.

### Clinical management and long-term follow-up

Postoperative care adopted a standardized management protocol: the distraction area was disinfected with alcohol and dressings changed every 2–3 days. Prophylactic oral antibiotics were administered for 1 week postoperatively to prevent infection, while closely monitoring pin tract and surrounding skin tissue conditions, observing for abnormal manifestations such as redness, swelling, or exudate. Patients were allowed weight-bearing ambulation to facilitate functional recovery, but special attention was paid to avoiding wound pressure.

Follow-up management employed a combination of remote and outpatient/inpatient modes: after discharge, dedicated medical personnel maintained regular contact with patients through online platforms, guiding rehabilitation training and understanding recovery conditions. If patients developed abnormal symptoms or complications, they could return to the hospital for treatment. Follow-up continued for 1 year after complete wound healing to ensure good long-term results.

### Clinical outcomes

Primary outcomes included the proportions of ulcer healing at 3 months and 6 months,the amputation rate, and the proportion of patients with wound healing recurrence within 1 year. Ulcers were considered healed when complete epithelialization with no drainage was observed and maintained for at least 2 weeks. Amputation at ankle joint level and below was defined as minor amputation; amputation above ankle joint level was defined as major amputation; recurrence was defined as wound recurrence 1 year after healing, whether at the original site or elsewhere [16].

### Statistical analysis

Due to the nature of the surgical interventions, neither the surgeons nor the data analysts were blinded to group allocation. In animal experiments, repeated measures ANOVA was used to analyze data changes across multiple time points or different measurement conditions for different treatment groups, followed by post-hoc pairwise comparisons. In clinical research, when comparing preoperative and postoperative experimental results, paired t-tests were used for analysis. Normality of demographic and clinical data was assessed using the Shapiro–Wilk test. For normally distributed variables, *t*-tests were used; for non-parametric variables, Mann–Whitney *U* tests were used; and for categorical data, chi-square tests or Fisher’s exact tests (if expected counts in any contingency cell were less than 5) were used for between-group comparisons. Continuous variables are presented as mean ± standard deviation, and categorical data are presented as counts and percentages. Statistical significance was set at *P* < 0.05. All statistical analyses were conducted using SPSS version 30.0.0 (IBM Corp, USA).

## Results

### Establishment of canine TPD animal model

**Fig. 1:**
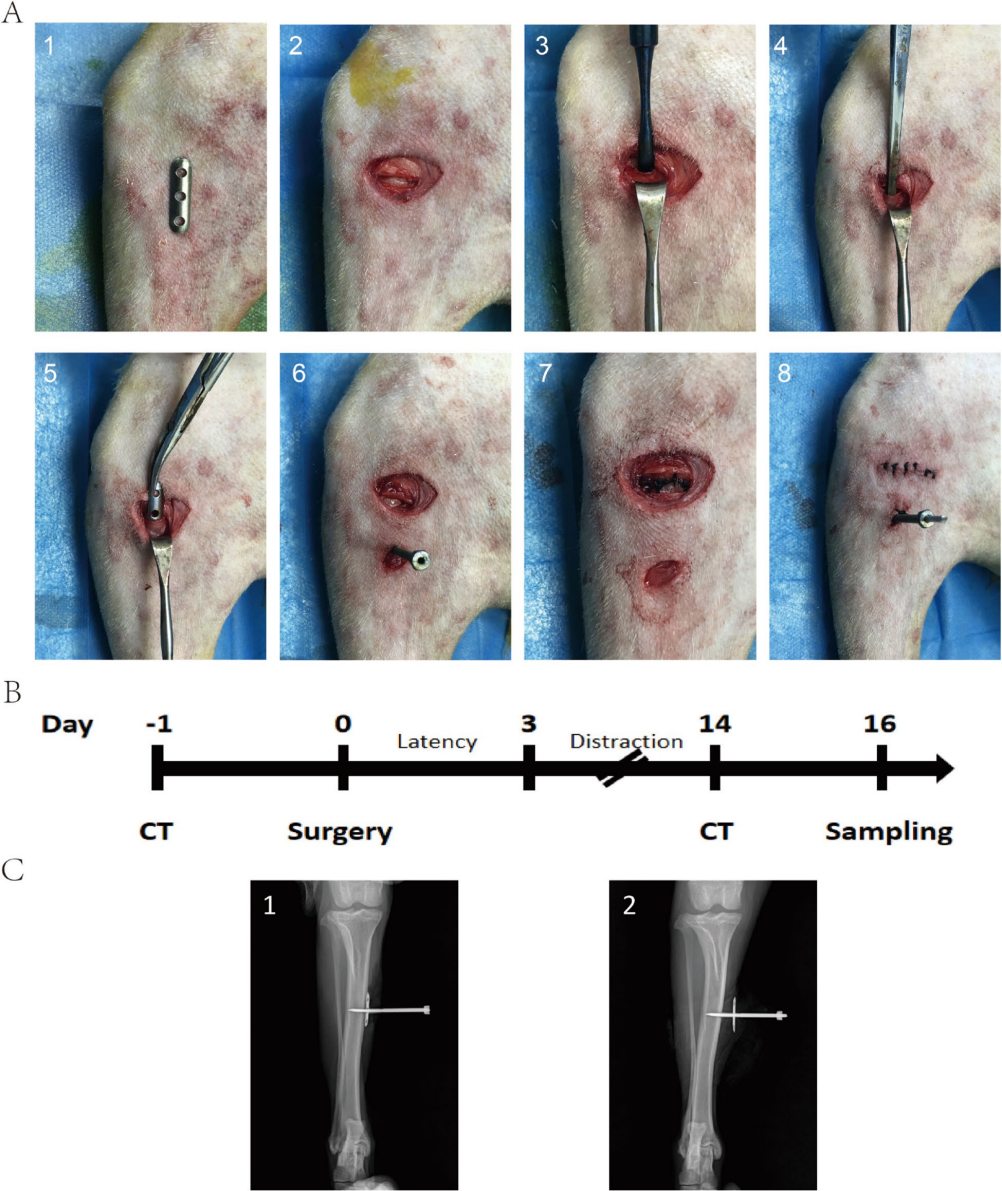
Establishment of Beagle dog TPD animal model. **A** TPD surgical procedure. **B** Experimental timeline. **C** Lateral tibial radiographs during TPD process. (1) Post-surgery before distraction. (2) Before removal of external fixation device at maximum distraction

After preoperative CT examination, TPD surgery was performed the next day. The surgical site was located at the medial flat area of the middle or proximal tibia. The distraction plate measured 0.5 cm × 2.5 cm with a thickness of 0.5 mm. The surgical procedure is shown in Fig. 1A. A smooth bone surface area was identified at the middle or proximal tibia, and a distraction plate was positioned on the skin surface to ensure no elevation (Fig. 1A1). A transverse incision was made at the proximal end of the distraction plate, with layer-by-layer separation of skin and subcutaneous tissue, exposing the tibial surface. Subsequently, the periosteum was forcefully incised transversely on the tibial surface, ensuring precise one-time incision to avoid multiple operations that could damage periosteal integrity (Fig. 1A2). A periosteal elevator was used to separate the periosteum toward the proximal and distal tibia along the incision line, separating the periosteum from the bone surface while ensuring periosteal integrity (Fig. 1A3). A bone file was inserted along the gap between the elevated periosteum and bone surface using the periosteal elevator, continuing to separate more periosteum from the bone surface. Care was taken to control force during separation to prevent the file from perforating the periosteum (Fig. 1A4).

The periosteum was elevated, and curved forceps were used to grasp the custom periosteal distraction plate, completely placing it between the periosteum and bone surface (Fig. 1A5). Cut through the skin and periosteum, insert the hollow screw into the threaded section in the middle of the distraction plate, and confirm whether successful docking can be achieved (Fig. 1A6). First remove the screw, then suture the proximal periosteum, soft tissues, and skin in layers (Fig. 1A7). Subsequently, dock the screw with the distraction plate again, use an electric drill to insert the Kirschner wire through the middle of the screw into the bone cortex, then cut off the excess Kirschner wire above, and suture the surrounding skin (Fig. 1A8). After the surgery, the screw was rotated clockwise again to observe successful rotation; if successful, it was reset. Following periosteal distraction device installation, a 15-mm dermatome was directly used on the ipsilateral foot to create a circular wound approximately 15 mm in diameter.

The distraction strategy is shown in Fig. 1B. After surgery completion, a 3-day rest period was observed to allow tibial wound skin growth, avoiding subsequent traction-induced tearing. Subsequently, distraction was performed at 0.5 mm per day for 11 days, with CT performed after TPD external fixation device removal on post-operative day 14. Successful distraction was determined through visual observation and lateral DR films (Fig. 1C).

### TPD promotes canine wound healing

**Fig. 2:**
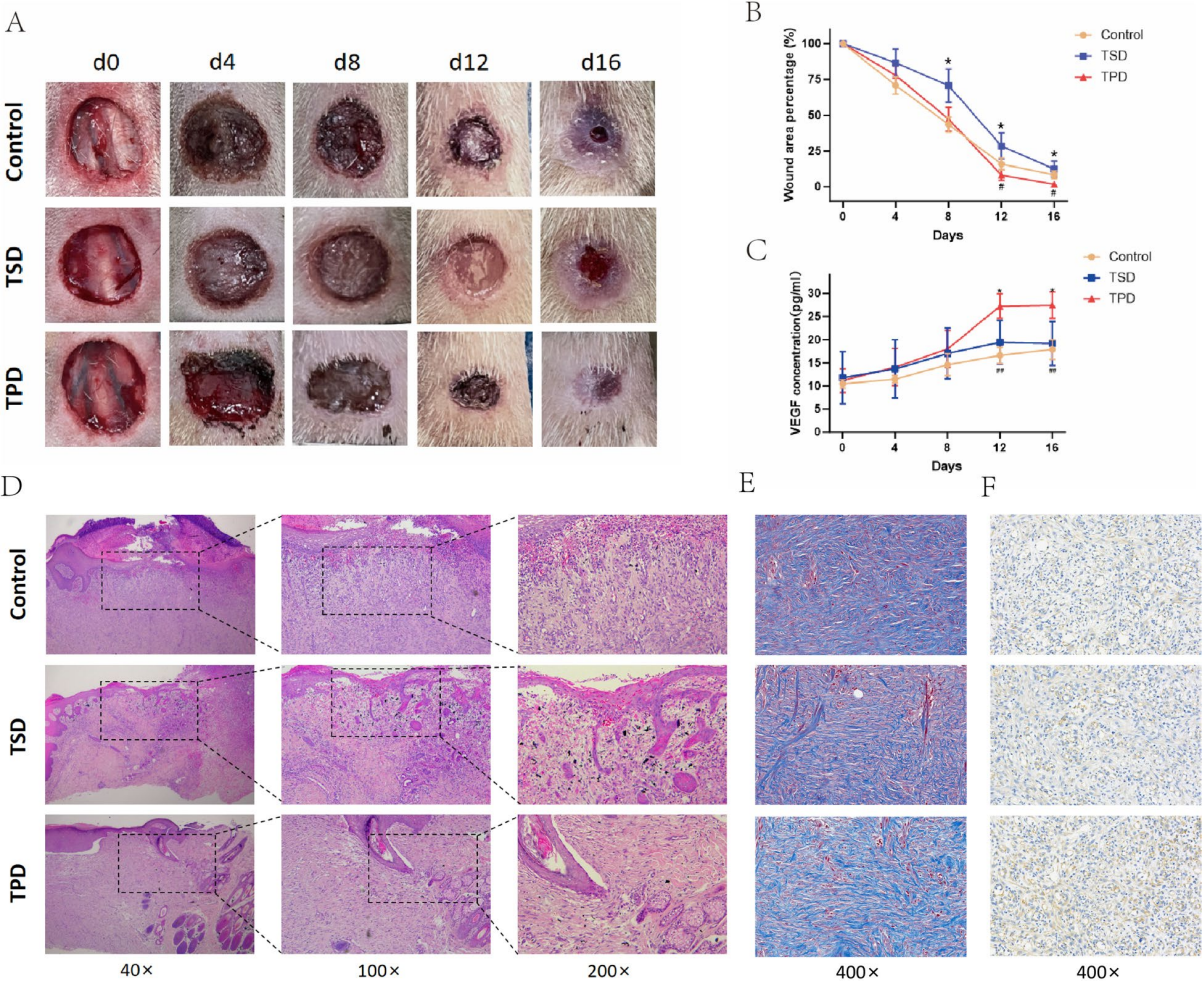
TPD promotes canine ILU wound healing.** A** Macroscopic appearance of wound healing time course (d0, d4, d8, d12, d16) for control, TSD, and TPD groups. **B** Percentage curve of wound area changes over time for each group. **C** Dynamic changes in VEGF concentration in canine blood for each group. **D** H&E staining of wound tissue (40 × , 100 × , 200 ×). **E** Masson staining of wound tissue (400 ×). **F** VEGF immunohistochemical staining of wound tissue (400 ×). *p < 0.05, TSD group vs TPD group; #*p* < 0.05, ##*p* < 0.01, control group vs TPD group

To evaluate treatment efficacy on wound healing, we systematically measured wound area changes in control, TSD, and TPD groups on postoperative days 0, 4, 8, 12, and 16 (Fig. 2A, B). Using initial wound area as baseline (100%), the TPD group demonstrated significantly accelerated healing in the mid-to-late period (days 8–16), achieving near-complete healing by day 16 (residual area 2.08 ± 1.68%), significantly superior to the control group (5.46 ± 1.98%) and TSD group (12.49 ± 2.97%, *P* < 0.05). Serum VEGF level monitoring by ELISA (Fig. 2C) revealed that while all groups showed comparable baseline levels (day 0, *p* = 0.97), the TPD group achieved peak VEGF expression at day 12 (27.25 ± 2.16 pg/ml), significantly higher than the TSD group (19.44 ± 3.85 pg/ml, *p* = 0.04) and the control group (16.61 ± 1.44 pg/ml, *p* = 0.002), maintaining elevated levels through day 16. This sustained high systemic VEGF expression in the TPD group closely correlated with accelerated wound healing. Immunohistochemical analysis of wound tissue VEGF expression (Fig. 2F) further confirmed enhanced local angiogenic factor production in the TPD group. Histological analysis (Fig. 2D, E) demonstrated that the TPD group exhibited superior tissue quality compared to other groups, characterized by an intact epithelial layer with appropriate thickness, compact subcutaneous tissue structure, significantly reduced inflammatory cell infiltration (HE staining), and regular collagen fiber arrangement with increased density (Masson staining).

### Changes in canine lower limb blood supply

**Fig. 3:**
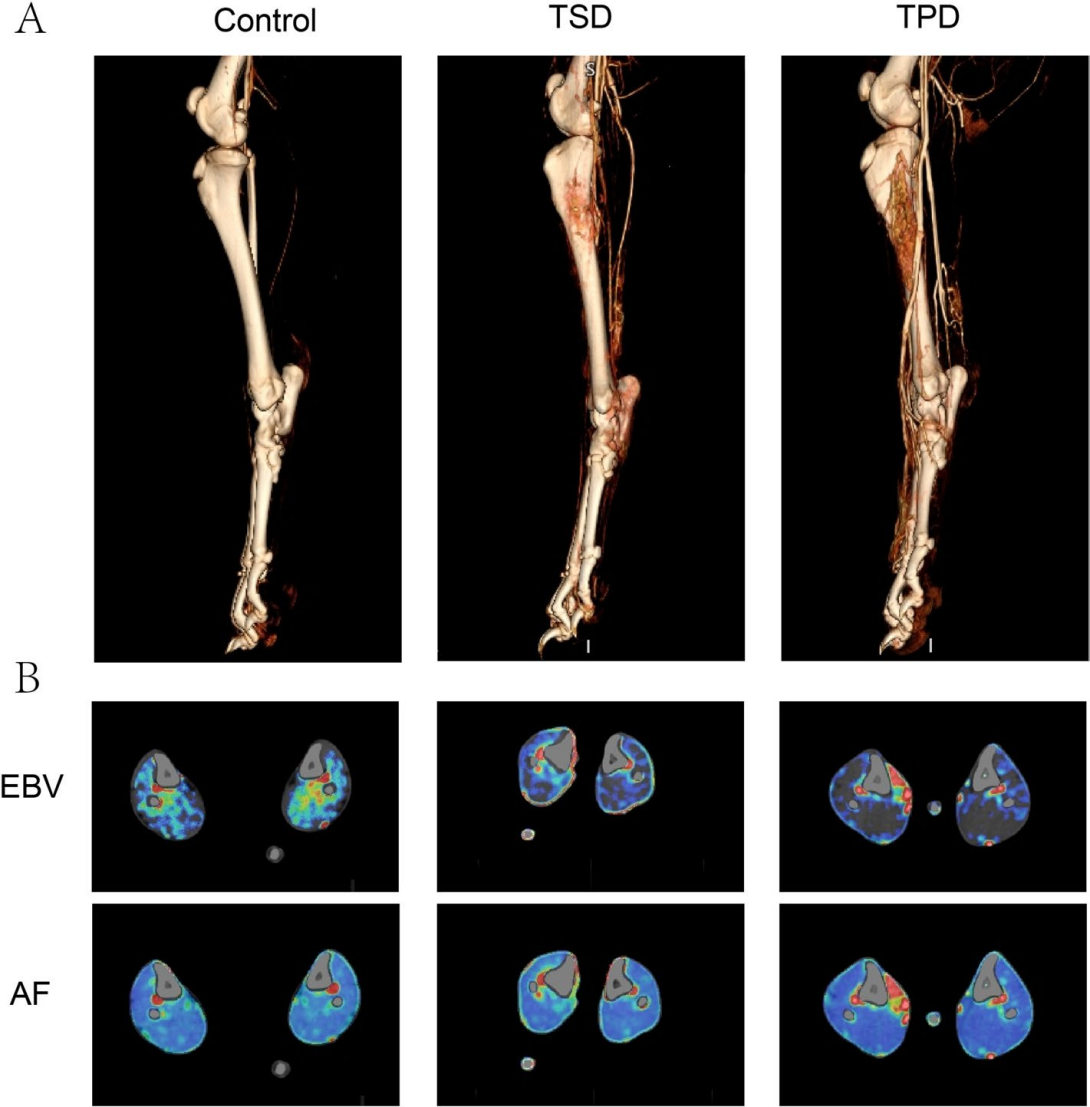
Canine lower limb CT examination 14 days post-surgery.** A** Canine lower limb CTA reconstruction image. **B** Canine lower limb CTP image. EBV representing the equivalent volume fraction of intravascular blood within a given tissue volume. AF indicating blood volume passing through tissue per unit time

CTA three-dimensional reconstruction images (Fig. 3A) showed that the control group had no visible microvascular network in the tibia, with generally normal lower limb vascular course but fewer branches. TSD group showed minimal vascular network at the distraction site, with slightly increased lower limb vascular opacification. TPD group formed dense vascular networks at the distraction site, with more abundant lower limb vascular branch opacification and more obvious collateral circulation formation, suggesting that TPD treatment promoted angiogenesis and collateral circulation establishment, benefiting lower limb blood supply improvement and wound healing. CTP parameter analysis (Fig. 3B) showed significant changes in perfusion patterns in TSD and TPD groups compared to control group. Morphologically, both TSD and TPD groups showed increased tissue volume at intervention sites, with TPD showing significantly higher tissue volume increase than TSD. Both TSD and TPD groups showed higher EBV and AF values in distraction regions, manifested as increased red areas, suggesting increased local blood volume, blood flow, and vascular permeability.

### Patient inclusion and exclusion

**Fig. 4:**
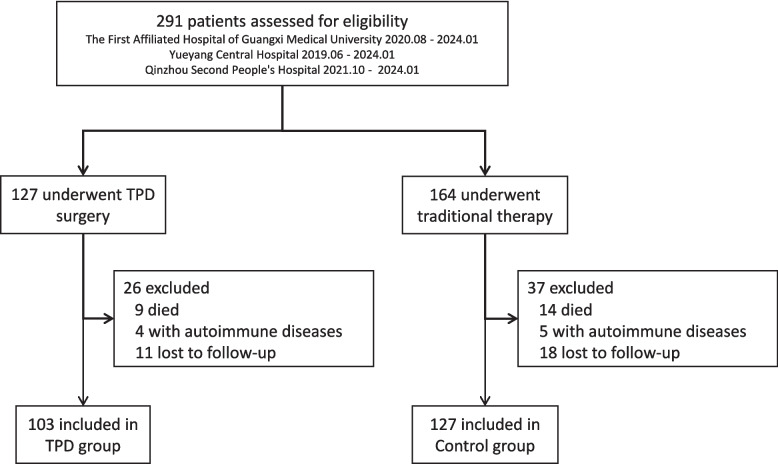
CONSORT flow diagram of patient enrollment and allocation

From June 2019 to January 2024, a total of 291 patients with ILU were assessed for eligibility at three participating centers: The First Affiliated Hospital of Guangxi Medical University, Yueyang Central Hospital, and Qinzhou Second People’s Hospital. Of these, 127 patients underwent TPD surgery and 164 received conventional therapy. After excluding patients who died during follow-up, those with autoimmune diseases, and those lost to follow-up, 103 patients in the TPD group and 127 patients in the control group were included in the final analysis (Fig. 4). Table 1 provides a detailed comparison of baseline demographic and clinical characteristics between the two groups. There were no differences between the TPD group and control group in terms of average age, gender ratio, body mass index (BMI), smoking history, and comorbidities. Regarding treatment protocols, except for the key difference of TPD intervention, preoperative and postoperative treatment protocols remained consistent between the two groups, further ensuring comparability of treatment measures. This balanced distribution of baseline characteristics not only eliminates potential confounding factors but also provides a reliable comparative basis for our subsequent evaluation of TPD intervention effects.

**Table 1:** Patient descriptive characteristics

Characteristics	TPD (*n* = 103)	Control (*n* = 127)	*P* value
Age (years)	64.3 ± 9.9	66.1 ± 11.1	0.585
Male sex, % (*n*)	72.8 (75)	78.0 (99)	0.367
Body-mass index (kg/m^2^)	24.6 ± 4.8	23.7 ± 5.1	0.163
Smoking, % (*n*)	39.8 (41)	47.2 (60)	0.258
Hypertension, % (*n*)	57.3 (59)	62.2 (79)	0.449
Coronary heart disease, % (*n*)	41.7 (43)	38.6 (49)	0.626
Dyslipidemia, % (*n*)	47.6 (49)	45.7 (58)	0.773
Chronic kidney disease, % (*n*)	19.4 (20)	14.2 (18)	0.287
Severe artery stenosis detected by CTA or ultrasound, % (*n*)	32.0 (33)	35.4 (45)	0.589
Diabetes mellitus, % (*n*)	85.4 (88)	78.7 (100)	0.191
Wagner classification			0.167
Grade 2	23.3 (24)	34.6 (44)	
Grade 3	48.5 (50)	40.2 (51)	
Grade 4	28.2 (29)	25.2 (32)	
Prior treatment, % (*n*)			
Revascularization	14.6 (15)	18.9 (24)	0.384
Debridement	28.2 (29)	34.6 (44)	0.293
Post treatment, % (*n*)			
Bone cement	35.9 (37)	43.3 (55)	0.256
Negative-pressure wound therapy	18.4 (19)	29.1 (37)	0.060
Skin grafting	7.8 (8)	10.2 (13)	0.518

Data are presented as the mean ± SD or % (*n*)

### Patient diagnosis and treatment protocol

**Fig. 5:**
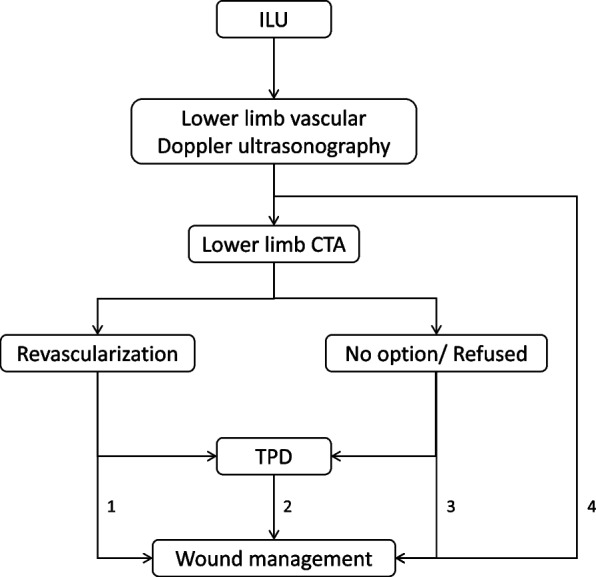
Patient admission diagnosis and treatment protocol. Patients were allocated to the TPD group (pathway 2) or control group (pathways 1, 3, and 4) based on whether they underwent TPD. All patients received standardized wound management

This study established a standardized diagnosis and treatment protocol for ILU patients (Fig. 5), ensuring all patients received systematic stepwise treatment. All admitted ILU patients first underwent bilateral lower limb vascular color Doppler ultrasound examination to assess the degree of lower limb vascular lesions and hemodynamic status. For patients with Doppler examination suggesting moderate to severe vascular stenosis or occlusion, lower limb CTA examination was further performed to obtain more precise vascular anatomical information. Based on imaging examination results, vascular surgery specialists evaluated whether patients were suitable for revascularization treatment. For patients with indications for vascular reconstruction, percutaneous vascular intervention or surgical vascular reconstruction was actively implemented. Patients were divided into the TPD group and the control group based on whether they underwent TPD surgery. The decision to perform TPD surgery was made according to patient preference after informed discussion of treatment options. All treatment decisions were based on multidisciplinary team discussions to ensure optimal treatment plans for each patient.

### Clinical TPD surgical operation, distraction strategy

**Fig. 6:**
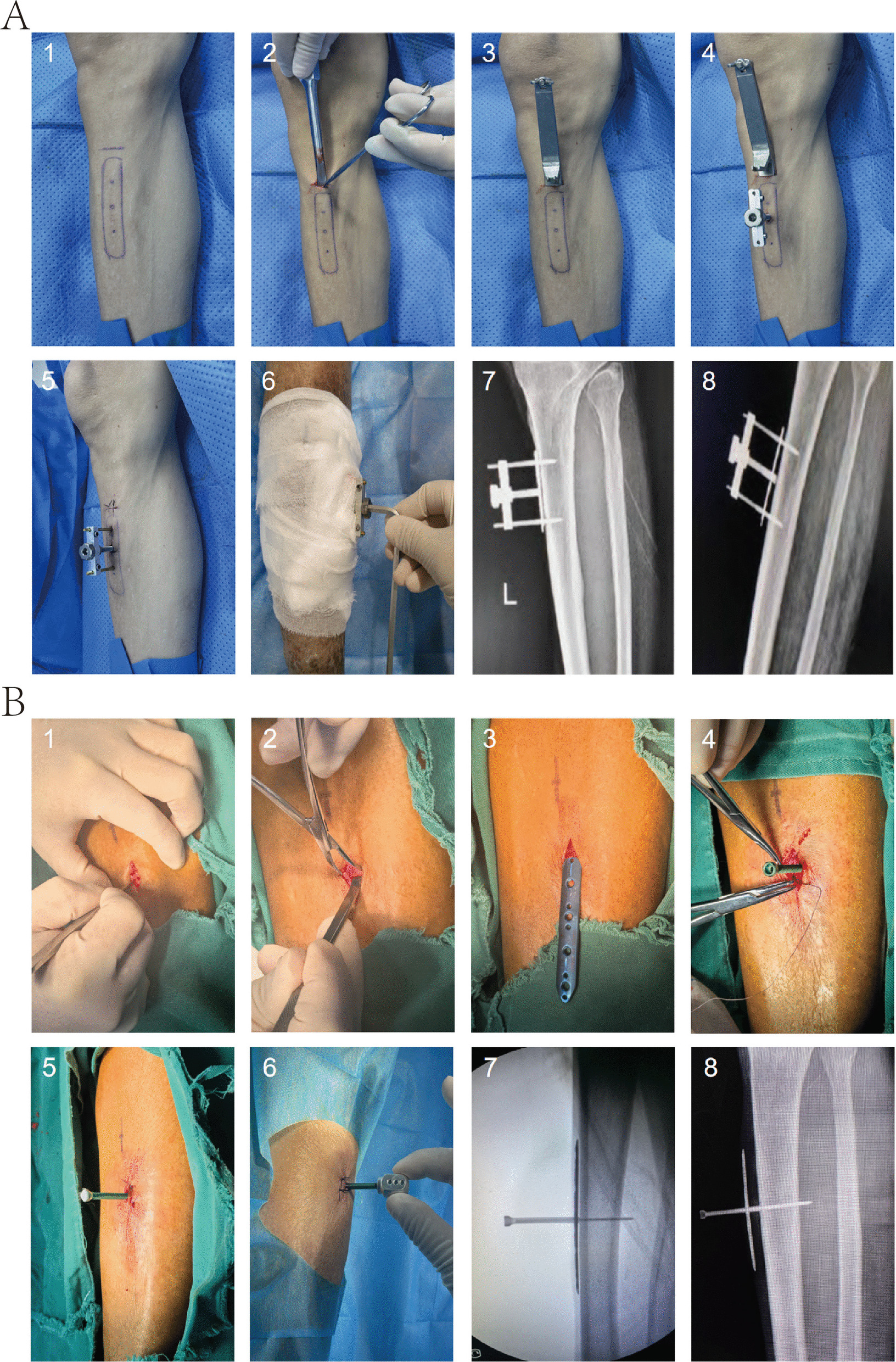
TPD surgical procedure flowchart. The surgical site was located at the anteromedial aspect of the tibia. (1) Determine surgical site, make incision through anteromedial tibial skin, soft tissue, and periosteum; (2) Use file to strip periosteum, separating periosteum from bone surface; (3) Lift periosteum, using gripping device (group A) or manually (group B) to implant distraction plate beneath periosteum on bone surface; (4) Install external fixation device; (5) Insert Kirschner wire for fixation. Kirschner wires can also be used as drills to create holes through the wound at other sites for medullary cavity decompression; (6) Use special tool to rotate screw during dressing changes, driving distraction plate elevation; (7) Lateral radiograph of periosteal external frame, distraction plate not yet elevated; (8) Lateral radiograph of periosteal external frame, distraction plate elevated to maximum height

Three centers in this study previously used two different surgical patents. Intraoperative procedures are shown in Fig. 6. A staged distraction protocol was implemented postoperatively: patients had a 3–5 day rest period after surgery, followed by titanium alloy plate distraction, with external fixation device adjustments every 6 h, each adjustment raising 0.25 mm, maintaining a constant daily distraction speed of 1 mm. After 10 days of continuous distraction, the maximum position was reached, with the distraction plate cumulatively elevated 10 mm. After completing the first phase of distraction, the distraction plate was readjusted to align with the bone surface and rested for 5 days. Subsequently, initiated the second phase of periosteal distraction, still at 1 mm per day distraction speed, continuing for another 10 days, elevating the distraction plate another 10 mm. At this point, the entire distraction process was complete, and the distraction device was removed under local anesthesia.

### Comparative analysis of clinical primary outcome indicators

**Fig. 7:**
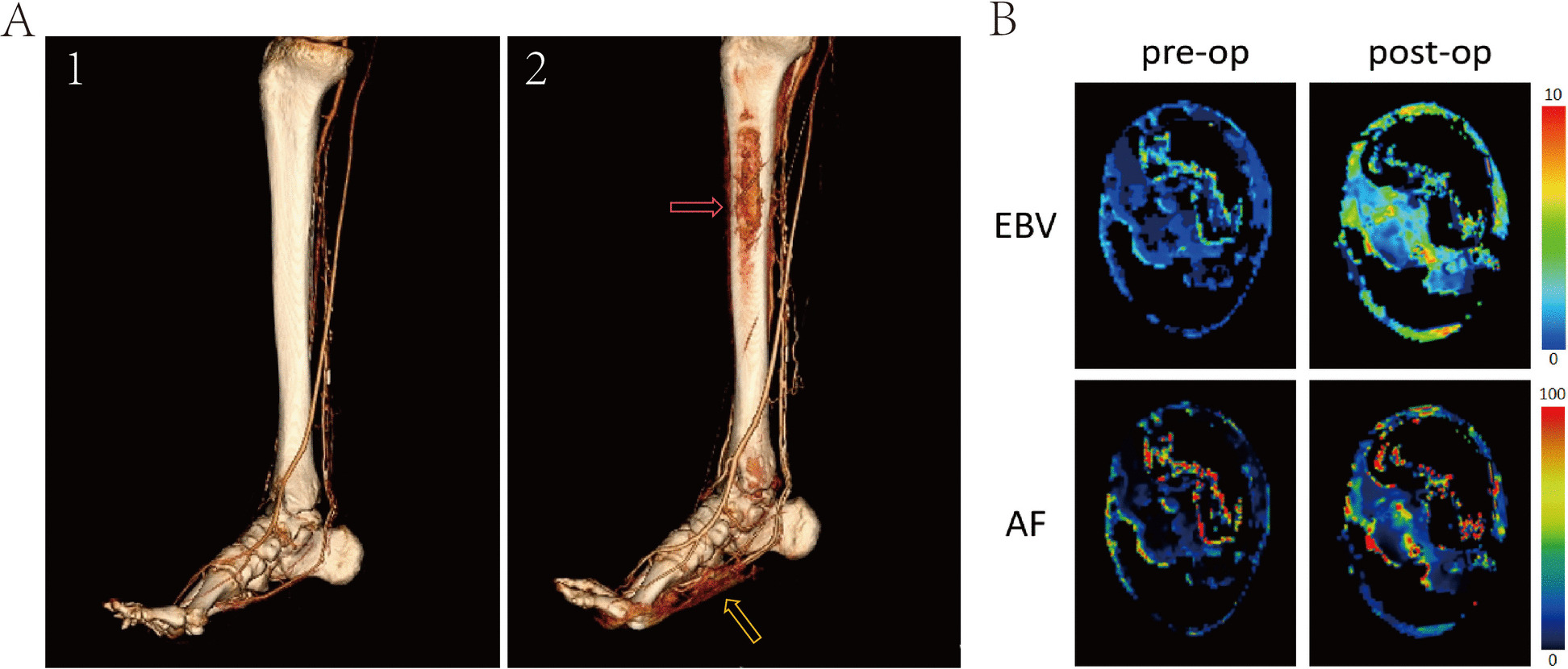
Pre- and postoperative lower limb imaging changes in patients. **A** Lower limb CTA. Postoperatively, dense microvascular networks (red arrow) can be seen at the surgical site on the medial aspect of the tibia, with increased vascular visualization (yellow arrow) in the foot. (1) Preoperative lower limb CTA. (2) Postoperative lower limb CTA after removal of the periosteal frame. **B** Axial CTP images of the foot. Postoperatively compared to preoperatively, EBV and AF significantly increased, with more complete foot visualization contours

**Fig. 8:**
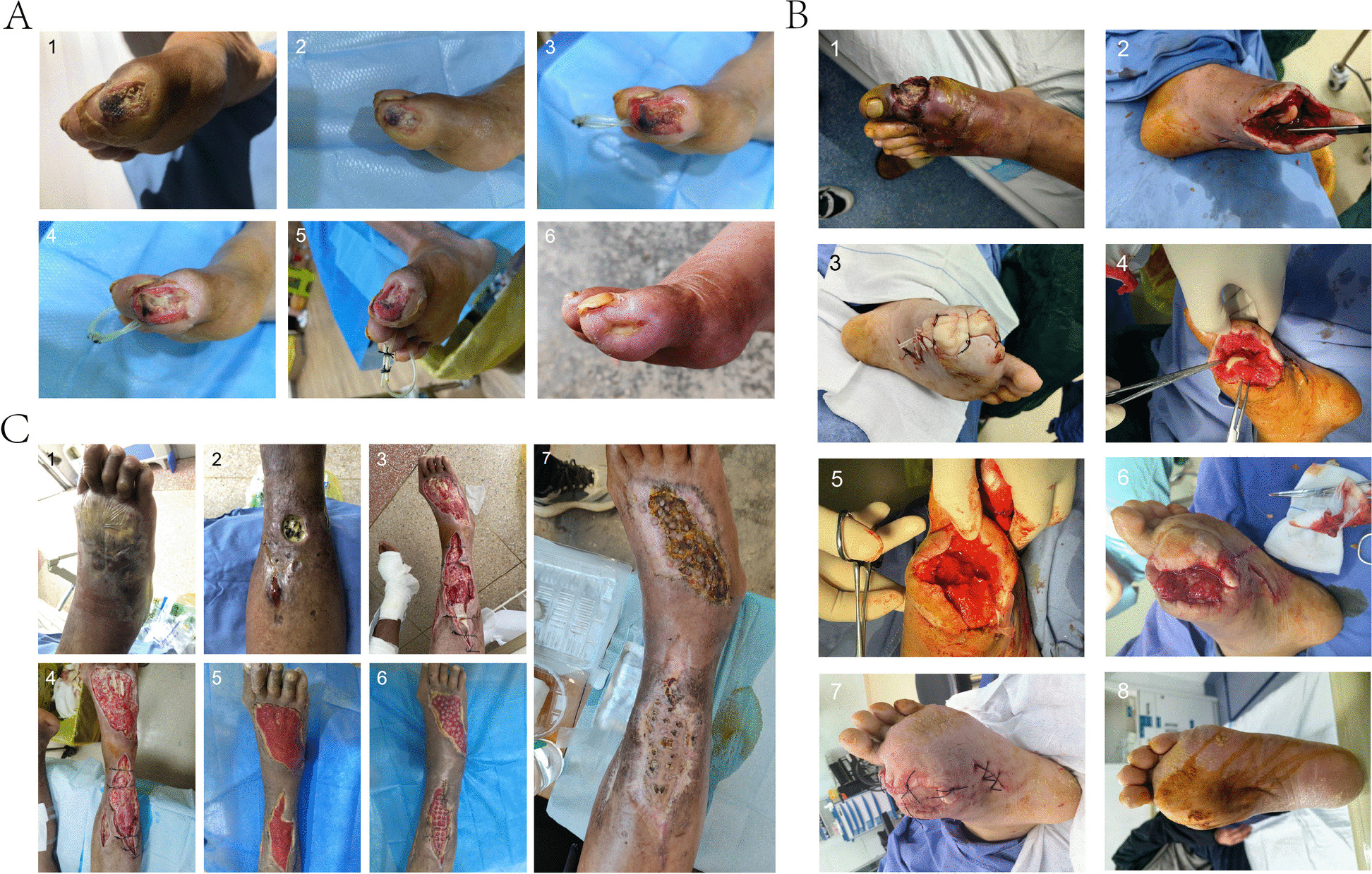
Representative cases of wound healing following TPD surgery. **A** (1) A diabetic foot patient presented with a traumatic ulcer that failed to heal after 2 months of treatment at an outside hospital. (2) After admission, debridement and wound dressing were performed; however, the wound remained infected and necrotic with no signs of healing after 1 month. (3, 4, 5) TPD surgery was performed. Postoperatively, granulation tissue growth was observed, but secondary superficial fascial necrosis occurred, requiring multiple debridements and dressing changes. (6) After wound necrosis resolved, the patient continued dressing changes at home. The wound healed 2 month after TPD surgery. **B** (1) A patient presented with diabetic foot complicated by mild arteriosclerosis obliterans. (2, 3) Based on intraoperative assessment, the great toe was amputated and bone cement coverage was applied. (4, 5) One month postoperatively, the patient returned for follow-up; necrotic bone tissue was removed and bone cement coverage was continued. (6, 7) Two months postoperatively, the patient returned for a second follow-up; debridement and bone cement coverage were continued. (8) The wound healed approximately 3 months postoperatively. **C** (1, 2) Patient with chronic non-healing wound for more than 1 year, complicated by acute infection, admitted to hospital. (3) Emergency debridement surgery, TPD surgery performed. (4) Within 1 week postoperatively, the patient’s wound surface showed abundant scattered purulent discharge. (5) One month postoperatively, the patient’s wound surface filled with fresh granulation tissue. (6) Punctate skin grafting performed on wound tissue. (7) More than 2 months after TPD surgery, the affected foot wound surface completely healed

Among the 103 patients in the TPD group, 93 patients achieved wound healing within 3 months postoperatively, and 97 patients achieved wound healing within 6 months postoperatively; among the 127 patients in the control group, 98 patients achieved wound healing within 3 months postoperatively, and 109 patients achieved wound healing within 6 months postoperatively. The TPD group showed superior wound healing rates compared to the control group, with 3-month wound healing rates of 90.3% vs. 77.2% (*P* = 0.008) and 6-month wound healing rates of 94.2% vs. 85.8% (*P* = 0.039). There was no statistically significant difference in minor amputation rates between the two groups, with the TPD group at 38.8% (40/103) and the control group at 44.1% (56/127) (*P* = 0.421). Regarding major amputation incidence, the TPD group was 2.9% (3/103) compared to the control group’s 9.5% (12/127), reaching statistical significance (*P* = 0.046). In the TPD group, 9 patients experienced recurrence within 1 year postoperatively, accounting for 8.7% of total cases; in the control group, 25 patients experienced recurrence within 1 year postoperatively, accounting for 19.7% of total cases. The recurrence rate in the TPD group was significantly lower than the control group (*P* = 0.020). See Table 2 for details. Figure 7 presents imaging changes before and after surgery in one patient, and Fig. 8 presents representative three cases of TPD treatment for ILU patients.

**Table 2:** Outcomes of patients

Characteristics	TPD (*n* = 103)	Control (*n* = 127)	*P* value
Ulcers healed by 3 months, % (*n*)	90.3 (93)	77.2 (98)	0.008
Ulcers healed by 6 months, % (*n*)	94.2 (97)	85.8 (109)	0.039
Minor amputation, % (*n*)	38.8 (40)	44.1 (56)	0.421
Major amputation, % (*n*)	2.9 (3)	9.5 (12)	0.046
Recurrence, % (*n*)	8.7 (9)	19.7 (25)	0.020

### Clinical complications and management

Among the 103 patients, 7 cases (6.8%) developed pin tract purulent discharge. Of these, 5 cases successfully controlled symptoms through suspension of distraction, enhanced wound dressing changes, and antibiotic treatment, and successfully completed the entire distraction process; 1 patient had to have the external fixation frame removed prematurely after several days of periosteal distraction; another case required debridement, and this patient’s wound gradually healed after removal of the external fixation frame and debridement surgery. During the distraction process, 7 patients reported severe pain, and 2 patients developed non-infectious skin erythema and swelling, which was considered to be due to distraction speed exceeding skin tolerance. For these 9 patients (8.7%), once problems were identified, the distraction plate was immediately repositioned to bone level, and after skin condition returned to normal, the distraction speed was adjusted to 0.5 mm/day, continuing distraction at this rate, and all eventually successfully completed the entire distraction process.

## Discussion

### Clinical challenges and treatment limitations in CLTI management

ILU is a severe stage of chronic limb-threatening ischemia (CLTI), which is a serious vascular disease that has become a focus of clinical attention in recent years due to its extremely high disability and amputation risk. Effective vascular reconstruction, including percutaneous vascular intervention and vascular bypass surgery, is a key strategy for limb salvage treatment of CLTI [16]. However, existing data show that up to one-third of CLTI patients cannot undergo effective vascular reconstruction due to complex lesions [17, 18]. For elderly patients or those with diabetes, their vascular lesions often involve extensive areas, affecting not only large vessels but also commonly involving small arteries and even capillary levels, presenting characteristics of diffuse microvascular disease [19]. Traditional vascular reconstruction mainly targets large vessel lesions and has a limited effect on improving peripheral microcirculation disorders. Persistent ischemia not only exacerbates local tissue metabolic disorders and nutritional deficiencies but also leads to significant immune function decline, thereby increasing the risk of secondary wound infection, delayed healing, and even recurrence after healing. This vicious cycle not only causes enormous physical suffering and an economic burden to patients but also significantly increases the necessity for subsequent amputation and the incidence of serious complications. TTT technology based on Ilizarov theory has shown encouraging clinical results in CLTI treatment in recent years [20]. However, this technology still faces the risks of bone malunion, fractures, and bone infections [21, 22]. Therefore, we attempted to develop a new treatment strategy that could achieve similar therapeutic effects while simplifying the operation process, reducing trauma, and lowering complication rates.

### Theoretical origins and evolution of TPD technology

Distraction osteogenesis technology, in addition to its mature application in orthopedic correction, has also achieved good results in oral and maxillofacial surgery for treating maxillofacial deformities [23]. To solve the common problem of insufficient bone volume in dental implant restoration, oral surgeons have actively explored the application value of periosteal distraction technology [24]. In 2002, Schmidt et al. first confirmed the phenomenon of new bone formation under the periosteum in rabbit mandibular periosteal distraction experiments, laying an important experimental foundation for the clinical translation of this technology [25]. Unfortunately, due to the relatively limited amount of new bone generated, which could not meet clinical needs, this technology could not be promoted in clinical practice, and related research was relatively scarce [26, 27]. It is worth noting that clinical orthopedic surgeons have deeply recognized the important role of the periosteum in fracture healing, vascular regeneration, and tissue repair processes through long-term practice. The periosteum is not only an important source of bone-forming cells but also plays a key regulatory function in angiogenesis and tissue remodeling. Based on these theoretical foundations and clinical observations, we hypothesized that TPD activate angiogenic responses in ischemic limbs through mechanisms similar to TTT technology, thereby promoting the healing process of ischemic wounds and reducing the recurrence rate. This hypothesis provides new insights for applying periosteal distraction technology to the treatment of CLTI patients.

### Experimental animal model selection

This experiment mainly targeted the tibial periosteum for intervention. In the selection of experimental animals, small animal models such as mice, rats, and guinea pigs are not suitable for this study because they are too small and their tibial periosteum is difficult to clearly identify with the naked eye. Among large animal models, although rabbits have appropriate body size, their periosteum is relatively thin, creating technical difficulties for experimental operations. Goats and pigs have appropriate body size, but their compliance is poor, especially the adjustment difficulty during the TPD process is significant with a high financial burden. Considering the animals’ body size, physiological characteristics, experimental operability, and economic factors comprehensively, this study ultimately chose dogs as experimental animals. In breed selection, Labrador dogs have larger body size, spacious tibias, and thicker periosteum, making surgical operations relatively easy, but their individual variability is large [28]. Beagle dogs have relatively smaller body size, and although surgical operation difficulty is slightly greater than Labradors, requiring more precise surgical instruments, and the lively and active nature of Beagles increases operational difficulty during adjustment, Beagles have the significant advantage of stable genetic traits, which can effectively reduce individual differences in experiments [29]. Based on the animal ethics requirement to minimize the use of experimental animals as much as possible, while considering the importance of reducing individual differences for the accuracy of wound healing speed, this study ultimately chose Beagle dogs as experimental subjects.

### TPD advantages and areas for improvement

TPD surgery has the advantage of being minimally invasive, with minimal damage to surrounding soft tissues and blood vessels during the operation, helping to maximize protection of local blood circulation and tissue repair capabilities [30]. Compared to other more complex vascular reconstruction treatments, patients have better tolerance for this procedure, especially suitable for elderly patients with serious internal medicine diseases who cannot tolerate traditional treatments such as vascular bypass grafting. TPD surgery is relatively simple to operate, with low cost and relatively small economic burden, and has low technical dependence on surgeons, making it easy to promote clinically. In addition, the external fixation frame used in TPD has a smaller volume, causing less restriction on patients’ daily life activities postoperatively, which may help improve patient postoperative compliance, thereby enhancing overall treatment effectiveness. At the same time, this technology does not involve bone disconnection or bone fracture remodeling processes, so theoretically the possibility of bone marrow infection and fracture is smaller, with no risk of postoperative bone non-union.

### Future research directions

Nine patients (8.7%) were unable to tolerate the pain caused by 1 mm daily distraction speed, but when we adjusted the distraction speed to 0.5 mm daily, their pain was significantly relieved and successful wound healing was achieved. This suggests that individualized adjustment of distraction speed may be an effective strategy during TPD treatment, but it also raises an important question: whether slower distraction speeds will delay or reduce wound healing effects or affect overall postoperative efficacy. Although TPD has demonstrated good clinical efficacy for treating CLTI patients, designing optimal distraction strategies (including distraction speed, intervals, and duration) while effectively controlling complications remains a key research priority. Furthermore, exploring the mechanisms underlying how periosteal distraction promotes wound healing is essential. Critical questions remain: What local changes occur at the periosteal distraction site? How are these changes transmitted to the wound? Which wound healing processes are specifically affected? Previous studies have shown that TTT increases blood osteopontin levels, promoting endothelial cell migration and wound healing [31]. Additionally, research has confirmed that exosomal miR-494-3p following TTT is effective in DFU treatment [32]. Whether these pathways produce similar therapeutic effects in TPD treatment of ILU remains an intriguing research question.

### Study limitations and potential biases

Although this study achieved positive results, there are some limitations. The sample size used in large animal studies was relatively small, which may affect statistical power; the canine animal model did not have lower limb ischemia, which differs from the extensive, chronic vascular occlusion in clinical patients and may affect the clinical translation value of research results. We mainly focused on whether lower limb blood flow increased after TPD and whether wound healing accelerated. Considering that we observed differences in the treatment groups, it appears that these limitations did not impact the core focus of the study. The clinical study was a retrospective study, and we could not accurately calculate the time of patient wound healing. To minimize patient recall bias as much as possible, we selected 3 months and 6 months postoperatively as follow-up time points. This follow-up method prevented us from making more detailed comparisons of wound healing speed between TPD and control group patients, limiting comprehensive assessment of TPD’s effect on promoting wound repair in CLTI patients. In this study, we used the Wagner classification for patients rather than the WIFI classification, which also made it impossible for us to compare our results with previous CLTI studies [33]. In addition, the hospitals participating in this study used two different surgical patents (Fig. 6), which may create some bias in complication statistics. Finally, although the results of this study showed that the minor amputation rate in the TPD group was lower than the control group (38.8% vs 44.1%), this difference did not reach statistical significance. We believe this may be related to two reasons: On one hand, amputation provides faster wound healing and functional recovery compared to limb salvage. Major amputation causes obvious limb loss and significant psychological impact, while minor amputation preserves limb appearance and function with better patient acceptance; on the other hand, the three hospitals involved in this study were all tertiary hospitals, with patients mostly transferred from lower-level hospitals after ineffective conservative treatment, which to some extent reduced opportunities for early intervention and toe preservation, having some impact on minor amputation statistics.

## Conclusions

This study demonstrated through animal and clinical trials that TPD has significant therapeutic effects on ILU, with mechanisms possibly closely related to promoting angiogenesis and collagen reconstruction. As a new technology with minimal trauma and simple operation, TPD may provide new options for clinical treatment of ILU, especially for patients who cannot undergo vascular reconstruction surgery, with important clinical application prospects.

## Data Availability

The original data can be obtained by contacting the first author.

## Abbreviations

AF: Arterial flow
CLTI: Chronic limb-threatening ischemia
CTA: CT angiography
CTP: CT perfusion
DFU: Diabetic foot ulcer
EBV: Equivalent blood volume
ELISA: Enzyme-linked immunosorbent assay
FE: Flow extraction product
ILU: Ischemic leg ulcer
PAD: Peripheral arterial disease
TPD: Tibial periosteal distraction
TSD: Tibial soft tissue distraction
TTT: Tibial transverse transport
VEGF: Vascular endothelial growth factor
